# The Anti-Neuroinflammatory Role of Anthocyanins and Their Metabolites for the Prevention and Treatment of Brain Disorders

**DOI:** 10.3390/ijms21228653

**Published:** 2020-11-17

**Authors:** Joana F. Henriques, Diana Serra, Teresa C. P. Dinis, Leonor M. Almeida

**Affiliations:** 1 CNC—Center for Neuroscience and Cell Biology, University of Coimbra, 3004-504 Coimbra, Portugal; joanafhenriques@gmail.com (J.F.H.); tcpdinis@ci.uc.pt (T.C.P.D.); malmeida@ci.uc.pt (L.M.A.); 2 Faculty of Pharmacy, University of Coimbra, 3000-548 Coimbra, Portugal

**Keywords:** anthocyanins, polyphenols, natural compounds, antioxidants, neuroinflammation, neuroprotection, brain disorders

## Abstract

Anthocyanins are naturally occurring polyphenols commonly found in fruits and vegetables. Numerous studies have described that anthocyanin-rich foods may play a crucial role in the prevention and treatment of different pathological conditions, which have encouraged their consumption around the world. Anthocyanins exhibit a significant neuroprotective role, mainly due to their well-recognized antioxidant and anti-inflammatory properties. Neuroinflammation is an intricate process relevant in both homeostatic and pathological circumstances. Since the progression of several neurological disorders relies on neuroinflammatory process, targeting brain inflammation has been considered a promising strategy in those conditions. Recent data have shown the anti-neuroinflammatory abilities of many anthocyanins and of their metabolites in the onset and development of several neurological disorders. In this review, it will be discussed the importance and the applicability of these polyphenolic compounds as neuroprotective agents and it will be also scrutinized the molecular mechanisms underlying the modulation of neuroinflammation by these natural compounds in the context of several brain diseases.

## 1. Introduction

Fruits and vegetables are important sources of nutrients, including minerals, vitamins, polyphenols, and dietary fibers, which have multiple relevant benefits in preventing and ameliorating several chronic diseases. Many of these benefits have been associated with antioxidant, anti-inflammatory, antimicrobial, and anticarcinogenic effects mediated by polyphenolic compounds present in these foods [1,2,3,4,5]. Thus, over the last years, the regular and high consumption of polyphenol-rich foods has been encouraged, particularly in the Western world [6,7,8]. In fact, berry extracts have shown significant health-promoting outcomes which have been largely attributed to a specific group of polyphenols called anthocyanins [9,10,11]. Anthocyanins are a large subclass of flavonoids, widely distributed in fruits and vegetables in the human diet. Among flavonoids, anthocyanins have gained prominence mainly due to their high intake in humans and their well-recognized antioxidant and anti-inflammatory activities [10,12], among others, making them promising agents for the prevention and treatment of distinct pathological conditions, such as cardiometabolic diseases, cancer, vision impairment, and neurological diseases [11,13,14,15,16,17]. Despite many evidences supporting that anthocyanins have the capacity to reach circulation and to exert their actions in peripheral tissues [18,19], this matter remains controversial and will be further discussed.

Worldwide, people have faced up to a continuous increase in life expectancy, mainly due to the technological improvements and medical advances in providing innovative approaches in prevention and life-sustaining therapies. Consequently, a substantial increase in the prevalence of aging-associated diseases has been reported and neurodegenerative diseases are one of the major challenges that modern health care system faces today, with no effective treatment yet. All brain diseases encompass the impairment of biological processes, such as immune and antioxidant responses [20,21]. These processes are believed to underlie the trigger of certain diseases, fostering the research on their intrinsic pathways and mechanisms. Conveniently, anthocyanins emerge as neuroprotective agents, acting as potential antioxidant compounds, by scavenging free radicals and contributing to an increase in endogenous antioxidants and/or to a decrease in endogenous reactive oxygen species (ROS) formation [12,16]. Additionally, evidences show that anthocyanins may modulate neuronal cell death signaling pathways [22,23], regulate mitochondrial function [24], inhibit protein aggregation, and potentiate autophagy [25], along with the ability to prevent excitotoxicity-induced neuronal cell death by maintaining calcium homeostasis [26]. Besides these action mechanisms, anthocyanins seem to have a beneficial impact on neuroinflammation, a key biological process implicated in the progression of several brain diseases [27,28]. The present review will collect and discuss the more recent data about the neuroprotective effects of anthocyanins in neurological disorders, focusing mainly on the anti-neuroinflammatory role of these polyphenolic compounds.

## 2. Anthocyanins

### 2.1. Sources, Subclasses, and Structural Features

Anthocyanins have attracted significant research interest because they are natural compounds with potential therapeutic properties, easily obtained in a normal diet. Fruits, particularly edible berries, are the main sources of anthocyanins, even though they are also present in spices, herbs, and red wine [29]. Anthocyanins are natural pigments that provide red, purple, or blue colors to several plant elements, acting as an important regulator of plant stability and quality in different environmental conditions [9,30]. Moreover, these polyphenolic compounds have been widely associated with a considerable inherent antioxidant activity, although more recently, it has been recognized that anthocyanins possess a vast range of beneficial effects in different biological processes, such as inflammation, apoptosis, excitotoxicity and protein homeostasis [31,32].

**Figure 1:**
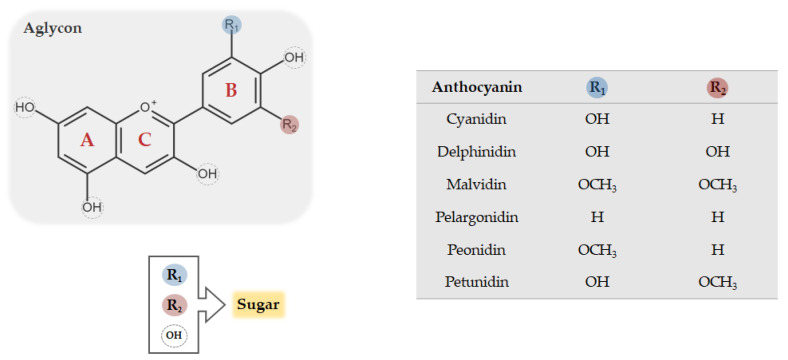
Chemical structure of common anthocyanins. The glycosidic form of anthocyanins is constituted by an aglycon, known as anthocyanidin, bounded to one or more sugar conjugates which may replace both R and OH groups.

Anthocyanins belong to the flavonoid class of compounds, presenting a typical chemical structure with two aromatic rings (A and B) coupled with three carbon atoms that form an oxygenated heterocycle ring (C) (Figure 1). The structural diversity of flavonoids depends on the number of possible combinations of substituents in its structure, i.e., hydroxylations, metoxylations, acylations, and mainly glycosylations. Anthocyanins occur in nature as glycosides, constituted by aglycones known as anthocyanidins. Figure 1 shows the basic structure of anthocyanins, the aglycon form, which can bind to one or more sugars in different positions, giving rise to distinct anthocyanin glycosides [19]. Anthocyanins are commonly separated in six naturally occurring different classes: Cyanidin, malvidin, delphinidin, petunidin, pelargonidin, and peonidin (Figure 1). There are several factors that underlie such differentiation, namely (1) the number and the position of hydroxyl and methoxyl groups; (2) the number and the position of sugars attached to the aglycon; and (3) the number and nature of aromatic or aliphatic acids that acylate anthocyanins [16]. In nature, there are about 700 structurally distinct anthocyanins and 27 different anthocyanidin molecules have been already identified [16].

Considerable research has revealed the structure-dependent properties of anthocyanins and the instigators of their structural changes [33]. Anthocyanins have a peculiar ability to modify their structure depending on the pH, acquiring unique antioxidant properties, distinct colors, and dissimilar stability at different pH values [34]. Due to these structural alterations and particularities, anthocyanins have an intrinsic electron deficiency, rendering them as strong antioxidant molecules with highly reactivity towards ROS. However, the capacity for ROS scavenging is not similar for all anthocyanins, being influenced by certain structural aspects, including the number and position of hydroxyl and methoxyl groups and their acylation and glycosylation levels. Additionally, the type of reactive species implicated also influences the efficacy of antioxidant activity of anthocyanins [16].

The relative abundance and the specific traits of anthocyanins significantly vary among different fruits and vegetables, depending on plant species and harvest conditions, and the general distribution of anthocyanins in major plant foods has been explored [35]. However, a wide disparity in the levels of anthocyanins ingestion has been reported by several authors [36]. This can be explained by the different evaluated regions and the subsequent social and cultural differences among populations. According to nutritional and educational backgrounds, the daily consumption of anthocyanins ranges from few milligrams to hundreds of milligrams [36]. Although it is not possible to define a reference daily intake of anthocyanins, the regular consumption of anthocyanin-rich fruits commonly leads to high, beneficial systemic levels of these compounds.

Over the last years, the impact of extraction and food processing techniques on natural anthocyanin’s contents and properties has been recognized [37,38]. In fact, the chemical stability of these compounds become compromised during extraction and food processing techniques, namely during thermal (e.g., drying, baking, and pasteurization) and mechanical (e.g., slicing and pressing) steps, as well as during unfavorable storage conditions [39]. These processes lead to smaller amounts of anthocyanins in final food products compared to raw material and, consequently, to the reduction of their beneficial properties [38], such as their potential neuroprotective role. Therefore, novel food processing technologies allowing the preservation of structural characteristics of anthocyanins should be further developed.

### 2.2. Pharmacokinetic Properties

The absorption of anthocyanins is influenced by their intrinsic physicochemical properties, i.e., by their structural characteristics and chemical reactivity, as well as by the region of gastrointestinal tract where absorption takes place. Anthocyanin absorption essentially occurs in the intestinal tract, although it has been suggested that the stomach could also be an important absorption site since the presence of anthocyanins or their metabolites in the plasma has been reported shortly after their ingestion [40]. Since anthocyanins are large molecules, which limits their passive diffusion, some studies have shown that anthocyanins probably cross the intact gastric mucosa barrier by active transport, which is facilitated by organic anion and glucose transporters, including bilitranslocase, glucose transporters (GLUTs) 1 and 3, organic anion transporter (OAT) 2 and sodium-coupled monocarboxylate transporters (SMCTs) 1 and 2 [41]. Otherwise, anthocyanin absorption may occur in the intestinal lumen either by active transport, involving multiple transporters expressed by intestinal epithelial cells, or to a lesser extent, by passive diffusion, possibly entailing the hydrolysis of anthocyanins to anthocyanidins [42]. The anthocyanin absorption from the different segments of small and large intestine is not only dependent on the molecular size and chemical structure of anthocyanins but also on food matrices, where physical and chemical interactions between compounds occur [43].

**Figure 2:**
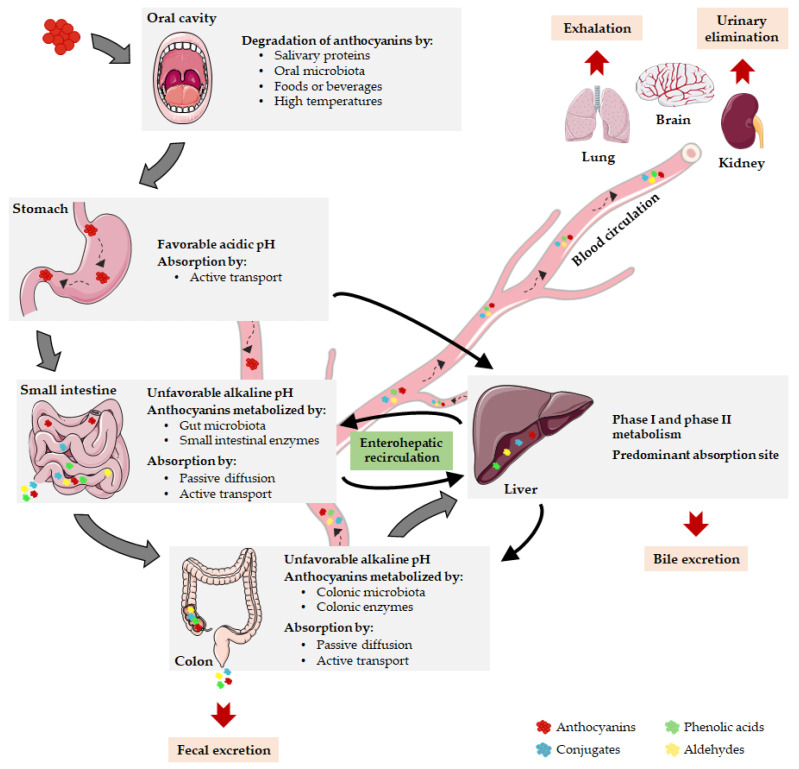
Pharmacokinetic properties of anthocyanins. After oral ingestion, anthocyanins can be degraded in oral cavity or reach the stomach, where they are stable due to gastric acidity. Here, they can be absorbed or can be delivered to the small intestine, undergoing metabolism or moving into the bloodstream. The remaining parent anthocyanins and intermediate metabolites transit through the small intestine into the colon where they are absorbed or extensively metabolized. Then, anthocyanins can be eliminated by fecal excretion or transported to the liver, the main absorption site. When parent anthocyanins or metabolites enter the blood circulation, they are distributed to target tissues, exerting their biological functions or being eliminated by exhalation, renal or bile excretion. Notably, anthocyanins can prevail during several days in the organism due to the enterohepatic recirculation.

The low absorption rates of anthocyanins reported by some authors can be attributed to anthocyanin degradation during gastrointestinal digestion [36,44,45]. At an initial stage of the digestion process, few studies proposed that anthocyanins can be degraded in the oral cavity due to several events, including the action of salivary proteins, the enzymatic activity of oral microbiota, the post-ingestion of foods or beverages and the high temperature of the oral cavity [46] (Figure 2). In contrast, the acidic gastric environment favors the stability of the glycosidic form of anthocyanins, and therefore their degradation in the stomach is unlikely to occur [43]. In the intestinal tract, it has been described that anthocyanins are extensively degraded by high pH levels and by the metabolic action of gut microbiota and intestinal enzymes [40] (Figure 2). The reduced absorption rate of anthocyanins can be improved by manipulation of their physicochemical properties, thus extending the retention time of these compounds in the upper part of the gastrointestinal tract or enhancing their stability during unfavorable conditions [47,48].

Similarly to absorption, the bioavailability of anthocyanins is estimated to be low, about 1% [43,44], and so only a small fraction of the ingested anthocyanins seems to reach the systemic circulation and the expected target organs. Several factors can contribute to this low rate, such as the apparent low anthocyanin absorption, the first-pass metabolism, the action of gastrointestinal microbiota and the pH fluctuations [16]. Also, the food matrix has a significant impact on the anthocyanin bioavailability, since the capacity of anthocyanins to be released from the food matrix depends on their structural diversity [36], as well as on the interactions between these compounds and food components [49,50]. In fact, the presence of other micronutrients and macronutrients in foods may also alter the absorption properties of anthocyanins [49]. Positive or negative synergistic interactions can occur between anthocyanins and coexisting compounds [37,49], which cooperate or compete for specialized transporters [50], or directly affect the stability of anthocyanins, ultimately impacting on the absorption and bioavailability of these polyphenolic compounds. For instance, the viscosity of food matrix affected the absorption of blackcurrant anthocyanins in rats [51], while the presence of alcohol in red wine reduced the absorption of anthocyanins in comparison with red grape juice [52]. Similarly, proteins in milk can interact with polyphenols and reduce their absorption accompanied by the decrease in their antioxidant capacity [53], demonstrating that food components actively interfere with the bioactivity of such compounds.

It is important to highlight that anthocyanin bioavailability may be greatly underestimated if only intact compounds are considered [54]. Anthocyanins and other flavonoids are subjected to several metabolic events, often resulting in high concentrations of metabolites in blood circulation as compared to their parent compounds [55,56]. Anthocyanins are metabolized either during their passage through gastrointestinal tract or in the circulatory system. Accordingly, the slight amount of intact anthocyanins excreted in urine suggests that they effectively undergo extensive metabolism [50]. The metabolism of anthocyanins mainly occurs in the enterocytes and involves intestinal and hepatic metabolic machinery. Anthocyanins are widely subjected to first-pass metabolism, deglycosylation and microbiota-mediated metabolic reactions, involving membrane transporters and several types of chemical reactions such as hydroxylation, conjugation, methylation, and glucuronidation [33,43]. The consequent metabolic products are directed to the liver, potentially undergoing biotransformation via phase I and phase II metabolic pathways [57]. The metabolites can return to the small intestine, enter the blood circulation or reach the large intestine that contains the vast majority of gut bacteria [58] (Figure 2). During the metabolic process, phenolic acids and aldehydes emerge as chemically derived metabolites of anthocyanins, including protocatechuic acid, vanillic acid, gallic acid, and phloroglucinol aldehyde [33]. These new compounds are not devoid of bioactivity and can even be more stable and active than their respective parent anthocyanins [32]. The amount of these metabolites increases distally through the gastrointestinal tract and the enterohepatic recycling significantly contributes to this phenomenon [48] (Figure 2). In fact, anthocyanins are capable of being taken up into enterohepatic circuit through their incorporation into bile after initial absorption and they can subsequently return from the liver to the intestine, where they are reabsorbed and return to the liver [33]. An extensive enterohepatic recycling of anthocyanins has been suggested by the substantial amount of anthocyanin metabolites found in human urine several days after the last administration [43]. Actually, it has been demonstrated that anthocyanins are repeatedly subjected to conjugation by bacterial and human enzymes, resulting in a wide range of anthocyanin phase 2 conjugates that recirculate in bile leading to an increase in anthocyanins residence time in vivo [48].

Anthocyanins and their metabolites are distributed differently throughout the body according to their unique uptake and absorption properties, having been found in different body compartments, such as plasma, eye, brain, liver, kidney, and lung [59,60,61] (Figure 2). Among the target tissues for anthocyanins distribution, the liver seems to be the predominant organ after anthocyanins absorption, potentially involving the bilitranslocase-mediated transport [36]. The brain is another relevant site for anthocyanins accumulation, since they have the capacity to cross the BBB and to reach several crucial brain regions, such as the cortex, hippocampus, striatum and cerebellum [36,62]. Therefore, anthocyanins can be envisaged as a promising approach to modulate brain disfunctions as it will be scrutinized later. Concerning to anthocyanin excretion, anthocyanins and their metabolites are eliminated in urine, feces, bile and breath (Figure 2). Shortly after the anthocyanins consumption, their elimination occurs preferentially in urine, remaining in feces over the subsequent 6–48 h after ingestion [43]. In fact, fecal bacteria may not entirely metabolize unabsorbed anthocyanins, resulting in fecal excretion of intact anthocyanins [48]. Moreover, renal elimination of anthocyanins and their metabolites is likely to occur and seems to entail the tubular secretion and a bilitranslocase isoform [43]. On the other hand, the intact and metabolized forms of anthocyanins also undergo significant bile excretion, in part regulated by enterohepatic recycling process, whereas volatile metabolites are expelled into the air via exhalation [50].

Since the bioavailability is still considered the Achilles’ heel of anthocyanins, numerous researchers have been focused on developing new approaches to improve the bioavailability and stability of these natural compounds [63,64,65]. Actually, the formulation and encapsulation of anthocyanins have been recognized as valuable strategies to overcome their bioavailability limitations by improving the control of anthocyanins release (time and location) and their protection from environmental conditions and from other food elements, decreasing their degradation rate and increasing their half-life [66]. Several formulation strategies, including liposomes, microspheres or nanoparticles, have been used to improve the bioavailability, stability and penetration across the blood–brain barrier (BBB) and, consequently, the therapeutic efficacy of these compounds [67]. Anthocyanins encapsulation in biodegradable nanoparticles, using polyethylene glycol-gold nanoparticles (PEG-AuNPs) or polylactide-co-glycolide (PLGA)-PEG as encapsulating agents, have exhibited neuroprotective effects and increased efficiency compared to unconjugated anthocyanins [64,68,69]. Moreover, anthocyanin-loaded chitosan nanoparticles have shown enhanced stability in a beverage model and delayed degradation rate in simulated gastrointestinal fluid when compared to free anthocyanins [66]. Other research groups showed improved stability and/or bioavailability of anthocyanins using diverse encapsulating agents, such as solid lipid nanoparticles [70], whey protein and citrus pectin biopolymers [71], and chondroitin sulfate polysaccharide [72]. The use of nanoemulsion and nanoliposome systems for the improvement of anthocyanin properties has also shown promising preliminary results, although future studies are still needed to improve the encapsulation efficiency with small particle sizes and the optimization of coating [65].

## 3. Neuroinflammation

Neuroinflammation normally encompasses a defense process aimed at restoring homeostasis after central nervous system (CNS) injury, involving both innate and adaptive immune systems. However, sustained neuroinflammatory response can lead to neuronal damage and/or death seen in many neurological diseases, such as Alzheimer’s disease, Parkinson’s disease, multiple sclerosis and autism [73]. Hence, both neurophysiological and neuropathological events are significantly promoted by neuroinflammation process, involving several signaling cascades and demanding various inflammatory mediators [74].

### 3.1. Cellular Mediators

Microglia and astrocytes are specialized glial cells that belong to innate immune system in the CNS, being responsible for the supervision of the brain microenvironment in physiological conditions. Activated microglia respond to foreign and endogenous damage signals by eliciting several immune responses, such as the release of inflammatory molecules and/or the phagocytosis of apoptotic or defective cells [75], while astrocytes essentially support neurons through the maintenance of homeostatic neuronal activity [76]. Following a disruption in CNS homeostasis, microglia activation precedes the activation of astrocytes, which induce the recruitment of microglia and other immune cells to the injury site [77]. The interplay between these non-neuronal cells is crucial upon CNS injury since microglia-derived inflammatory factors may stimulate astrocytes to intensify the inflammatory response or to induce neurons to react for its survival [76,78]. However, these interactions may become detrimental, leading to an uncontrolled inflammatory state in case of microglial overactivation due to an insufficient astrocyte suppressive role or to an exacerbated astrocyte activity, which elicit the production of excessive amounts of pro-inflammatory and cytotoxic molecules [79]. In fact, the dysregulated activation of microglia may provoke the excessive release of pro-inflammatory cytokines, ROS and glutamate, triggering neurodegenerative mechanisms, which can contribute to serious neuronal damage [80].

Neuroinflammation is a complex process that not only relies on the participation of glial cells but also on the contribution of recruited peripheral immune cells [77]. Upon CNS homeostatic imbalance, the glial cells reactivity [75] and the subsequent release of pro-inflammatory mediators [80] may result in BBB disruption and increased permeability, allowing the infiltration of peripheral leukocytes into the brain. The peripheral inflammatory cells comprise monocytes, neutrophils, T and B cells, whose involvement differs among CNS diseases. Actually, these peripheral cells amplify the immune response in the CNS by producing more inflammatory molecules, which can eventually contribute to CNS disease progression [81]. Taken together, to understand the precise role of the immune cells and the molecular mechanisms underlying the neuroinflammatory response is crucial to identify relevant molecular targets or to develop effective therapeutic strategies to restore CNS homeostasis.

### 3.2. Signaling Molecules and Molecular Mechanisms

The neuroinflammatory response, comprising CNS-resident cells, peripheral immune cells, and signaling molecules, is triggered by several factors, such as infection, brain injury, autoimmunity or aging [73]. During neuroinflammation, functional phenotype acquired by microglia is intrinsically associated with the activation of microglial receptors, which elicits distinct intracellular pathways depending on the extracellular signals and on their corresponding receptors [82]. Bacterial lipopolysaccharide (LPS) is a typical initiator of various signal transduction cascades, which binds to the toll-like receptor (TLR) 4 in microglia, activating multiple signaling pathways and promoting diverse inflammatory events, including microglial phagocytosis and the release of inflammatory molecules [83]. Besides LPS, these inflammatory pathways are also induced by diverse ligands of other pattern-recognition receptors and of cytokine receptors, such as tumor necrosis factor (TNF) receptor [54,61].

The intracellular signaling pathways involved in neuroinflammation comprise the phosphoinositide 3-kinase/protein kinase B (PI3K/Akt) pathway, the toll-like receptor 4/myeloid differentiation primary response 88/nuclear factor kappa B (TLR4/MyD88/NF-κB) signaling cascade pathway, the Janus kinase/signal transducer and activator of transcription (JAK/STAT) pathway, the mitogen-activated protein kinases (MAPKs) cascades, among others [24,73]. The complex interaction between these neuroinflammatory pathways leads to an intricate signaling network, involving coordinated cellular responses that become dysregulated in chronic inflammation [84]. The activation of neuroinflammatory pathways occurs following the binding of extracellular stimuli (cytokines, growth factors, hormones, insulin, LPS, etc.) to cell surface receptors, activating many intracellular signaling proteins. The resulting signal transduction cascade culminates in the activation of transcription factors whose function is to mediate the production of pro-inflammatory cytokines, chemokines and cytotoxic molecules [73].

Among the transcription factors that are activated during neuroinflammation, the nuclear factor kappa B (NF-ĸB) emerged as a pivotal signaling molecule due to its ability to control the gene expression of many important pro-inflammatory molecules, including nitric oxide synthase (iNOS), cyclooxygenase 2 (COX-2) and cytokines. Additionally, NF-ĸB also modulates the proliferation, function and survival of the cells involved in both innate and adaptive immune responses [85,86]. In homeostatic conditions, NF-κB remains in its inactivated state, in the cytoplasm, forming complexes with IκB inhibitory family members or IκB-like proteins. The activation of NF-κB occurs in neuronal and non-neuronal cells through the canonical or the non-canonical pathways [87]. In the canonical NF-κB pathway, the inhibitory molecule IκBα is bounded to RelA/p50 heterodimer, constituting the passive form of NF-κB protein. The degradation of IκBα induced by IĸB kinase (IKK) molecule allows the translocation of the RelA/p50 to the nucleus, where it binds to specific DNA elements inducing the upregulation of inflammatory genes [85]. On the other hand, the non-canonical NF-κB pathway involves the activation of specific signaling molecules, NF-ĸB-inducing kinase (NIK) and IKK, that mediate the p100 dissociation of the NF-κB complex, resulting in the nuclear translocation of the RelB/p52 heterodimer and in the subsequent induction of immune and inflammatory responses [87]. Even though the NF-κB activity is controlled by NF-κB inhibitors, this transcription factor appears to function concomitantly with other transcription factors, since they are activated by the same stimuli and regulated by the same signaling transduction cascades. Actually, some studies have reported the cooperative action of NF-κB and activator protein 1 (AP-1) transcription factors [88], as well as the simultaneous activation of c-Jun N-terminal kinase (JNK) and NF-κB through the involvement of intermediate signaling molecules [89]. Also, the crosstalk between NF-κB and Forkhead box O3a (FOXO3a) seems to promote microglial survival in oxidative stress conditions [90].

PI3Ks are enzymes found inside the cell contiguous with the plasma membrane. Following a variety of external signals, such as cytokines or LPS, several downstream molecules become activated, triggering the PI3K signaling pathway [91]. During this intracellular signaling cascade, a number of intermediate molecules activate PI3K which, in turn, promotes Akt activation. The phosphorylation of Akt regulates several target proteins implicated in various biological activities, including cell survival, apoptosis, protein synthesis and inflammatory responses [92]. The PI3K/Akt signaling pathway also involves the contribution of mammalian target of rapamycin (mTOR) protein kinase which increments Akt activity and/or stimulates NF-κB activity, promoting the upregulation of inflammatory genes [91]. The downregulation of PI3K/Akt-dependent signaling pathways, that culminates in the activation of target proteins such as NF-κB, mTOR or FOXO1, has been related to the inhibition of LPS-induced neuroinflammation [92,93,94]. Therefore, these signaling pathways appear to be relevant as therapeutic targets for microglia-mediated neuroinflammation.

Microglia activation also elicits MAPKs activation, a family of protein kinases that includes p38 kinase, c-Jun N-terminal kinases (JNK1, 2, 3) and extracellular signal-regulated kinases (ERKs). Both p38 and JNK kinases are activated by environmental stress and inflammatory factors, while ERKs are usually triggered by mitogens and growth factors [95]. These MAPK signaling pathways roughly consist in three intermediate proteins that are successively activated: firstly, MAPK kinase kinase (MAPKKK) activates MAPK kinase which, subsequently, phosphorylates and activates MAPK [96]. Upon MAPK activation, several target proteins, such as AP-1, NF-ĸB and c-jun transcription factors, are activated and regulate the transcription of many inflammatory mediators [73]. For instance, MAPK pathways have been demonstrated to control iNOS and cytokines production in LPS- and cytokine-activated glial cells, showing the crucial role of these signaling pathways in the regulation of neuroinflammation [24].

The neuroinflammation response also relies on the activation of JAK/STAT pathway, which occurs following the binding of cytokines, growth factors and related molecules to their respective receptors. The activation of these receptors coupled to JAK promotes the phosphorylation of STAT proteins which dimerize and translocate to the nucleus to induce the transcription of inflammatory genes [96]. In astrocytes, it has been described that the inhibition of STAT-1 phosphorylation compromises the production of inflammatory molecules [24]. On the other hand, JAKs mediate the activation of MAPK and PI3K pathways, resulting in the further activation of transcription factors associated with pro-inflammatory outcomes [97]. In fact, there is a close interplay between these signaling pathways illustrated by STATs ability to induce the activation of distinct transcription factors or cofactors that are also modulated by other neuroinflammatory signaling molecules [98]. Thus, all these interactions demand the integrative understanding of the consequences of the aberrant activation of these mechanisms and the viability of manipulation of such interconnected neuroinflammatory pathways as a potential protective therapy for neuroinflammatory disorders.

## 4. Anti-Neuroinflammatory Activity of Anthocyanins and Their Metabolites

The neuroinflammation plays a crucial role in the development of many brain disorders and data gathered from in vitro and in vivo studies have shown that anthocyanins can significantly reduce the chronic inflammatory state in such pathological conditions (Table 1 and Table 2). For this reason, anthocyanins have emerged as potential dietary neuroprotective agents for brain diseases. Numerous reports have addressed the neuroprotective role of anthocyanins, despite a few data on the anthocyanin-mediated regulation of brain inflammatory responses have been gathered. This section will focus on how these phenolic compounds can be suitable therapeutic agents for CNS disorders, targeting the neuroinflammatory pathways underlying brain’s innate immune system.

As mentioned before, LPS exposure is one of the most conventional methods to study microglia activation in vitro. Therefore, several in vitro studies have been using LPS-activated microglia as a model of microglia driven neuroinflammation to obtain deep understanding of the molecular mechanisms underlying microglial responses during the neuroinflammatory process [82]. Poulose et al. revealed that anthocyanin-rich açai fruit pulp fractions protected BV2 microglial cells exposed to LPS, concomitant with a significant suppression of p38 and NF-κB activation, and a decrease in iNOS, COX-2 and TNF-α levels [28]. Moreover, another study demonstrated that anthocyanins, extracted from black soybean seed coats, prevented the LPS-induced activation of NF-κB, PI3K/Akt and MAPKs signaling cascades in BV2 microglial cells, reducing the production of pro-inflammatory mediators, including nitric oxide (NO), prostaglandin E2 (PGE2), TNF-α and interleukin (IL)-1β [99]. Likewise, the anthocyanin callistephin has been demonstrated to be involved in the regulation of iNOS, TNF-α and NF-ĸB in LPS-treated C8-4B microglial cells, potentially via suppression of p38 phosphorylation [100]. In agreement with these results, a very recent study confirmed the ability of cyanidin-3-O-glucoside (C3G), the most common anthocyanin subfamily, to prevent the LPS-stimulated BV2 microglial cell activation, via inhibition of NF-κB and p38 MAPK pathways, suppressing the production of pro-inflammatory mediators, such as NO, PGE2, IL-1β, and IL-6 [101]. However, in another study, C3G demonstrated to be unable to abolish LPS-induced NO production in BV2 microglia cells [121]. The disparity of these results might be explained by differences in experimental conditions, namely the distinct C3G concentrations and treatment periods used in such studies. Also, protocatechuic acid (PA), a key metabolite of anthocyanins, has been recognized as a neuroprotective agent, showing antioxidant and anti-inflammatory properties in different pathological circumstances. Wang et al. demonstrated that PA inhibits the TLR4-mediated NF-ĸB and MAPKs pathways, halting the subsequent release of pro-inflammatory mediators such as TNF-α, IL-6, IL-1β, and PGE2 in LPS-stimulated BV2 microglia cells [102]. Accordingly, other findings showed that PA seems to be able to reduce NO production in LPS-activated BV2 microglial cells through unknown mechanisms, while 4-hydroxybenzoic acid (HBA), another anthocyanin metabolite, did not change the LPS-increased NO levels [103]. On the other hand, Vafeiadou et al. reported that different concentrations of pelargonidin had no significant effect on LPS/interferon (IFN)-γ-induced production of TNF-α and NO in glial cells [122]. The anti-inflammatory activity of anthocyanins or their metabolites is known to depend on their structural features; however, their structure-activity relationships are not yet fully understood [12,123,124]. This could explain why HBA and pelargonidin are not able to influence the neuroimmune response.

The systemic administration of LPS in animals has been generally used to study microglia-mediated neuroinflammation in vivo. In adult mice treated with Korean black soybeans-derived anthocyanin, the LPS-induced increase in p-NF-ĸB, IL-1β and TNF-α levels was attenuated in the cerebral cortex, with a simultaneous decrease in cortical astrocytosis and microglia activation [108]. In line with these data, anthocyanin extracts from *Vaccinium myrtillus* L. prevented memory deficits in LPS-exposed mice, producing similar neuroprotective effects, which included the restoration of IL-1β, TNF-α, and IL-10 levels in the hippocampus, the reduction of microglia and astrocyte activation in both cortex and hippocampus, accompanied by the inhibition of peripheral immune cells infiltration in the same brain areas [27]. Similarly, Khan et al. reported the ability of anthocyanins from soybean seed coat to decrease the hippocampal levels of inflammatory markers, namely p-NF-κB, TNF-α, and IL-1β, and to improve memory impairment in LPS-treated mice, also postulating that these beneficial effects occur via downregulation of JNK pathway mediated by anthocyanins [109]. Taken together, these in vivo studies strongly corroborate the efficient regulation of brain inflammatory responses by anthocyanins which have been also reported in in vitro studies, although the underlying mechanisms are not fully elucidated.

In the last few years, the modulation of microglial phenotype has gained growing interest since downregulating the neuroinflammatory response of microglia has shown a beneficial impact on neuronal survival [125]. Interestingly, the shifting of microglia polarization, also accepted as microglia phenotype reprogramming, has emerged as an appealing therapeutic approach in diverse pathological backgrounds [126,127,128]. In fact, the controlled switching between microglial pro- and anti-inflammatory phenotypes as an alternative to the complete blockage of microglial activation might be a more reasonable and precise treatment for some brain diseases [126]. However, targeting microglial phenotypic switch is still a puzzling approach, since the plasticity of microglia allows them to shift between a range of phenotypes instead of acquiring a strict and individual activation state [129,130]. In this context, a recent study revealed that naringenin, a natural grapefruit flavonoid, was able to shift the pro-inflammatory microglia phenotype to an anti-inflammatory state in LPS-stimulated BV2 cells. This naringenin-promoted switching of microglia polarization was dependent on inhibition of JNK signaling pathway, involving the downregulation of pro-inflammatory markers, such as TNF-α and IL-1β, and the upregulation of anti-inflammatory markers, such as IL-4, IL-10, and arginase-1 [131]. These results suggest the potential ability of flavonoids to exert their neuroprotective role through the modulation of microglia polarization shifting. However, regarding anthocyanins, Meireles et al. showed that C3G and a methylated form of C3G can attenuate pro-inflammatory markers and modulate microglia-neuron communication even though they are unable to shift LPS/IL-4 stimulated microglia to an anti-inflammatory state [104]. Therefore, further research is required to clarify whether anthocyanins can effectively target brain inflammation via modulation of microglia polarization state, creating new therapeutic avenues for neuroinflammatory diseases.

### 4.1. Brain Aging and Perioperative Neurocognitive Disorders

Aging is characterized by gradual biological changes that commonly lead to general functional deterioration and to severe neuropathological conditions. Remarkably, several findings have evidenced a beneficial role of anthocyanin-rich fruit-based diets in an aging context [32,132]. Blueberry-rich diet exhibited beneficial effects on motor and cognitive declines in different aged rat models, involving an increase in neuronal signaling and a decrease in brain oxidative and inflammatory mediators [133,134,135,136]. In accordance with these animal studies, a six-year trial showed that a higher long-term ingestion of berries and flavonoids seems to revert cognitive decline in older women, suggesting that a berry-supplemented diet can be truly advantageous for the improvement of the cognitive function [137].

D-galactose (DG)-induced accelerated aging model is recurrently used for induction of neuropathological alterations similar to human brain aging, promoting oxidative stress and inflammatory response which result in cognitive dysfunction and neurodegeneration [138]. Anthocyanins, extracted from Korean black soybean, have been reported to abolish microglia and astrocyte activation and abrogate neuroinflammatory response by suppressing NF-ĸB activation, leading to the reduction of iNOS and TNF-α levels in the hippocampal and cortical regions of DG-treated rats [110]. Similarly, Chen et al. also revealed the efficacy of anthocyanins from *Lycium ruthenicum* Murr. in alleviating memory dysfunction of DG-exposed rats, along with the ability to inhibit both microgliosis and astrocytosis, and to mitigate the activation or overexpression of NF-ĸB, COX-2, IL-1β and TNF-α in hippocampus [111]. Using the same aging-model, protocatechuic acid has shown to reduce the production of IL-1β, TNF-α, IL-6, and PGE2 in whole brain lysates, as well as to significantly decrease the activity of COX-2 and NF-ĸB following DG treatment [112]. Overall, these in vivo studies truly suggest that the control of neuroinflammatory responses, often accompanied by the attenuation of oxidative stress, apoptosis and neuronal dysfunction, can represent a promising strategy to hamper the neuropathophysiological process that underlies brain aging.

Perioperative neurocognitive disorders (PND) are characterized by cognitive decline before and/or after surgery similar to that found in neurodegenerative disorders [139]. Studies demonstrate that elder people are more susceptible to develop PND [139,140], thus pinpointing the influence of age in cognitive decline. The correlation between PND and neuroinflammation has been considered, since surgery promotes the development of a neuroinflammatory response that involves microglia activation and the production of inflammatory molecules [141]. Interestingly, a pilot study showed that pomegranate juice, containing substantial anthocyanins concentration, was able to provide a long-term protection against PND-induced memory deterioration in humans [142]. Accordingly, anthocyanins isolated from *Lycium ruthenicum* Murr. were administered to adult mice subjected to a surgical procedure, promoting the amelioration of learning and memory abilities after surgery-induced cognitive impairment [113]. In addition, the anthocyanins treatment significantly attenuated microglia activation and neuroinflammation in hippocampus, along with the prevention of mixed-lineage protein kinase 3 (MLK3) activation, an upstream signaling molecule of JNK and p38 MAPK cascades [113]. Although neuroinflammation is essential to PND development, other risk factors may contribute to cognitive dysfunction, which often persists days or months after a surgical intervention, with significant consequences for the patient health status [141]. Importantly, due to the relevance of the neuroinflammatory response caused by surgical events or by pre-existing inflammatory pathologies on PND onset, it is imperative to identify potential neuroinflammation-targeted agents. In this context, polyphenolic compounds, particularly anthocyanins, rise as strong candidates to mitigate PND complications in patients subjected to surgical procedures.

### 4.2. Alzheimer’s Disease

Alzheimer’s disease (AD) is a recurrent neurological disorder caused by amyloid beta peptide (Aβ) accumulation and hyperphosphorylation of tau protein in the brain, culminating in memory and cognitive deficits. Although numerous mechanisms have been reported to underlie the AD development [143], the cause of this multifactorial neurodegenerative disease is not well understood yet. Innate immune response is a well-known contributor of AD onset, since misfolded proteins bind to neuroimmune cells, triggering the release of inflammatory mediators which further promotes the disease progression [144]. Thus, targeting these neuroinflammatory mechanisms appears as a beneficial strategy to control the pathogenic process in AD.

The potential therapeutic value of anthocyanins for delaying AD progression have been explored [45]. In fact, prospective studies analyzed the impact of flavonoids intake in cognitive dysfunction in older adults, showing that cognitive decline decreases with the increase in berries intake in older women [137]. Also, the consumption of strawberries and other flavonoid-rich foods may decrease the risk of Alzheimer’s dementia [145]. Beyond the well explored antioxidant properties of anthocyanins, several studies have emerged focusing on the anti-neuroinflammatory role of anthocyanins and their metabolites in the context of AD. Indeed, Aβ-induced neuroinflammation and tau agglomeration are recognized as critical events for the overt pathological features in APP/PSEN1 mouse model for AD [146]. Accordingly, an in vitro study showed that a blueberry supplementation, rich in anthocyanins, suppressed p44/42 MAPK-dependent pathway in primary microglia, resulting in the reduction of microglial inflammation, inhibition of Aβ aggregation and increment of microglial clearance of Aβ [147]. The authors also proposed that these data could explain the prevention of behavioral deficits in APP/PSEN1 mouse model [148]. Fully activated microglia are associated with Aβ clearance [149,150], while partial microglial activation is implicated in neurodegeneration [151]. Using the same double transgenic mice, Li et al. observed that bilberry anthocyanins induced the full activation of microglia and astrocytes, also regulating the mediators of synaptic and phagocytic functions of those cells, which include the triggering receptor expressed on myeloid cells (TREM2), TLR2/4, C-X3-C chemokine receptor 1 (CX3CR1), and tyrosine kinase binding protein (TYROBP) [114]. Also, bilberry anthocyanins improved memory and cognitive functions, by reducing LPS brain levels and downregulating inflammatory mediators in hippocampus, namely NF-ĸB, COX-2, iNOS, TNF-α, IL-1β, IL-6, and CD33 [114]. These results substantiate that bilberry anthocyanins consumption improve microglial Aβ clearance, since microglia play an essential role in the engulfment of fibrillar Aβ.

Anthocyanin metabolites have also been studied in the context of AD progression. In an AD mouse model, a grape seed extract rich in polyphenols, including gallic acid, attenuated Aβ deposition in the brain, with a concomitant decrease in microgliosis [152]. Interestingly, the observed reduction of senile plaques was associated with enhanced microglial phagocytic activity [152]. Accordingly, gallic acid treatment also efficiently counteracted the Aβ-induced cytokine release in both BV2 and primary microglial cells, through the inhibition of NF-ĸB activation, preventing the Aβ-mediated neurotoxicity and neuronal cell death [105]. The same authors showed that adult mice co-treated with Aβ peptide and gallic acid exhibited amelioration of the impairments in Aβ-induced learning and memory, along with the decrease in iNOS, COX-2, TNF-α, and IL-1β levels in hippocampus and cortex [105]. Similarly, Song et al. reported that protocatechuic acid significantly reduced the levels of TNF-α, IL-1β, IL-6, and IL-8 in the brain of APP/PSEN1 double transgenic mice, in parallel with the improvement of the learning and memory abilities and the attenuation of Aβ accumulation [115]. Moreover, in TNF-α-challenged C6 glial cells, delphinidin has shown to be able to downregulate two major inflammatory markers, the monocyte chemoattractant protein 1 (MCP-1) and the cytokine-induced neutrophil chemoattractant 1 (CINC-1) [106]. Unexpectedly, delphinidin had no effect on γ-secretase enzymatic activity, which is important for Aβ plaques accumulation [106]. The absence of a direct impact on this enzymatic activity has been supported by studies showing no alterations on Aβ production and deposition after blueberry-supplemented diets [148], or by the lack of blueberry polyphenols impact on the metabolism of amyloid precursor protein [153]. Although further research is required to gain mechanistic insights into AD onset, these results indicate that the neuroprotective role of anthocyanins in amelioration of AD may rely on their antioxidant and anti-neuroinflammatory properties, rather than on the direct effect on Aβ plaques formation and accumulation.

The poor bioavailability and absorption of anthocyanins may be limiting factors which can hamper their effective application in several pathological conditions. Therefore, the incorporation of anthocyanins into carrier systems can be useful to attain a long-term circulation and as a delivery system to the target site. In this sense, several efforts have been made to improve and to implement these approaches in AD therapy. Anthocyanins encapsulated in biodegradable nanoparticles based on PLGA and PEG displayed neuroprotective effects, via p38/JNK signaling pathway in Aβ_1-42_-treated SH-SY5Y cell line, accompanied by the inhibition of p-NF-κB, TNF-α, and iNOS expression [68]. Similarly, anthocyanin-loaded PEG-AuNPs showed higher efficacy, compared to unconjugated anthocyanins, against Aβ_1-42_-induced neuroinflammation and neurodegeneration, via p-JNK/NF-κB/p-GSK3β pathway, in both in vitro and in vivo AD models [64]. The same research group also reported that anthocyanin-loaded PEG-AuNPs were more effective than free anthocyanins in preventing the hyperphosphorylation of tau protein by regulating the p-PI3K/p-Akt/p-GSK3β signaling cascade, in addition to ameliorate memory impairments and to inhibit apoptosis and neurodegeneration in the Aβ_1-42_ mouse model of AD [69]. Nonetheless, in order to enhance the biological activities of anthocyanins, including their anti-neuroinflammatory role, the controlled and targeted release of anthocyanins along with their modification should be worthy of attention in the scope of CNS diseases.

### 4.3. Parkinson’s Disease

Parkinson’s disease (PD) is a progressive neurodegenerative disease characterized by loss of dopaminergic neurons and protein accumulation inside the remaining neurons, along with motor impairments. Among the mechanisms underlying PD progression, chronic neuroinflammation likely contributes to the pathophysiological process, which is evidenced by microglia activation, release of neurotoxic mediators and inflammatory-induced oxidative stress [154]. Therefore, targeting neuroinflammatory events seems to be a suitable therapeutic approach in the context of PD. A substantial number of studies have explored the potential neuroprotective activity of naturally occurring flavonoids, including anthocyanins, in PD onset [155]. Accordingly, a prospective study revealed that lower risk of developing PD in humans was associated with higher anthocyanins intake, particularly in men [156]. Shukitt-Hale et al. elegantly demonstrated that aged rats consuming berry-supplemented diets, including a substantial amount of anthocyanins, exhibited improved motor performance and increased hippocampal neurogenesis [157]. Findings from this research group and others suggest that the anti-neuroinflammatory properties of these natural compounds may contribute to ameliorate the PD symptoms [28,135,136,158].

LPS-treated mice has been extensively used as a PD model, since various observations revealed that LPS may induce the progressive loss of dopaminergic neurons and motor impairments analogous to the clinical symptoms of PD in humans, which are often correlated with immune dysregulation and the inherent molecular and cellular mechanisms [159,160]. Oral administration of purple sweet potato colors, seen as stable anthocyanins, seemed to be effective in improving the motor disabilities in LPS-treated mice. These findings were related with the ability of those anthocyanins to suppress the acute inflammatory response through the inhibition of JNK, ERK and NF-κB signaling pathways, preventing the upregulation of iNOS and COX-2 and the overproduction of TNF-α, IL-1β, and IL-6 in mice brain [116]. Nevertheless, the lack of studies comprising long-term consumption of isolated anthocyanins targeting neuroinflammation in PD context, does not allow to fully assess the anthocyanins effectiveness in preventing neuroinflammation-associated damaging. In fact, the main findings have been related to antioxidant and anti-apoptotic activities of anthocyanins in PD development [155]. Moreover, other polyphenolic compounds, including luteolin [161], apigenin [162], theaflavin [163], and resveratrol [164], have exhibited anti-neuroinflammatory activity in different PD models, implying the anti-neuroinflammatory role of anthocyanins in the same pathological conditions.

### 4.4. Multiple Sclerosis and Myelin Dysfunction

Multiple sclerosis (MS) is a neuroinflammatory disease caused by the degeneration of demyelinated axons exposed to oxidative stress and inflammatory harmful effects. Hence, the lack of effective therapies for MS lesions encouraged the search for antioxidant and anti-inflammatory agents that could contribute for the delay of MS onset and progression [165]. The neuroprotective role of anthocyanins, extracted from commercial grape skin, has been described in a demyelinating model using ethidium bromide (EB) [117]. Here, anthocyanins were able to reduce the inflammatory cells infiltration triggered by EB-induced demyelination, along with the restoration of TNF-α, IL-6, IL-1β, IFN-γ, and IL-10 levels in demyelinated rat pons [117]. These findings are in agreement with another study showing the ability of wine ingredients, which include anthocyanins, in promoting developmental myelination in an in vitro embryonic mouse model [166]. On the other hand, Siddiqui et al. explored the neuroinflammatory potential of both gallic acid (GA) and vanillic acid (VA) in an in vitro lysolecithin (LPC)-induced model of demyelination, in which hippocampal neurons were co-cultured with glial cells and subsequently treated with LPC [107]. Both anthocyanin metabolites were efficient in reducing COX-2 and NF-κB expression, in addition to significantly decrease the glial fibrillary acidic protein, a marker of reactive astrocytes [107]. Moreover, the same authors showed that GA and VA not only inhibited LPC-induced neuronal demyelination but further promoted myelin formation from immature oligodendrocytes, attributing this neuroprotection to the anti-neuroinflammatory activity of both compounds [107]. Taken together, these data support the hypothesis that anthocyanins and their metabolites could be considered promising therapeutic agents for MS, which could be used as adjuvants to the conventional therapies or possible candidates for clinical trials due to their positive effect in reducing local brain inflammation in demyelinated lesions.

### 4.5. Ischemic Stroke

The increased consumption of fruits and vegetables has been recognized as a beneficial dietary practice, protecting against certain pathological states, including ischemic stroke. A few studies have demonstrate the potential role of flavonoid subclasses, such as anthocyanins, in lowering ischemic stroke susceptibility due to their neuroprotective role [43]. A prospective study in human adults revealed that a higher anthocyanins intake contributed to a blood pressure decrease, resulting in about 12% reduction in propensity for hypertension, a well-known risk factor for stroke [167]. However, a further study from Cassidy et al. indicated that anthocyanins have only a slight impact in diminishing the risk of total and ischemic stroke in women [168]. Even though the pathophysiological mechanisms underlying stroke onset are multifactorial, inflammation appears as an evident contributor and the anti-neuroinflammatory activity of anthocyanins has been explored in the context of brain ischemia. Safaeian et al. demonstrated that C3G extracted from *Echium amoenum* petals was efficient in reducing myeloperoxidase activity, an indicator of leukocyte infiltration, in the brain tissue following cerebral ischemia induced by bilateral common carotid arteries occlusion/reperfusion in rats [169]. In a similar study involving a middle cerebral artery occlusion/reperfusion as an animal model of ischemia, anthocyanins isolated from *Myrica rubra*, mainly C3G (roughly 95%), were given to mice for seven days prior to surgery [118]. The authors showed that this anthocyanin extract were able to decrease the protein expression levels of TLR4, TNF-α, NLR family pyrin domain-containing protein 3 (NLRP3) and IL-18 in cerebral cortex, preventing the inflammatory response associated with these signaling pathways and suggesting that TLR4 might be an additional target for anthocyanins [118]. Also, the potential neuroprotective role of vanillic acid against pathological alterations in adult rats subjected to two-vessel occlusion and subsequent reperfusion has been reported. The pre-treatment with vanillic acid for 14 consecutive days allowed to mitigate memory deficits induced by cerebral hypoperfusion-reperfusion, as well as to recover the hippocampal levels of pro-inflammatory cytokines (IL-6 and TNF-α) and of the anti-inflammatory cytokine IL-10 [119]. Pan et al. used an anthocyanin derivative, namely pentunidin-3-O-rutinoside (p-coumaroyl)-5-O-glucoside, extracted from *Lycium ruthenicum* Murr., to protect against brain ischemia in rats subjected to middle cerebral artery occlusion/reperfusion [120]. Even though this phenolic compound exerts its protective role by several mechanisms, its administration markedly decreased infarct volume and cerebral edema, accompanied by the reduction of the cortical levels of inflammation-related molecules, namely TNF-α, IL-1β, and IL-6, also correlating the NF-κB and NLRP3 inflammasome pathways with these noticeable anti-neuroinflammatory effects [120]. These results confirm the anti-inflammatory potential of anthocyanin metabolites and raise the possibility that other similar compounds could be promising candidates to prevent the progression of ischemic injury. In this context, a recent review of Manolescu et al. discusses the results of epidemiological, in vitro, in vivo, and clinical studies that demonstrate the beneficial effects of anthocyanins and their metabolites in the vascular endothelium biology and their potential preventive use in cardiovascular disease, including stroke [43].

## 5. Gut Microbiota Impact on Anti-Neuroinflammatory Activity of Anthocyanins

**Figure 3:**
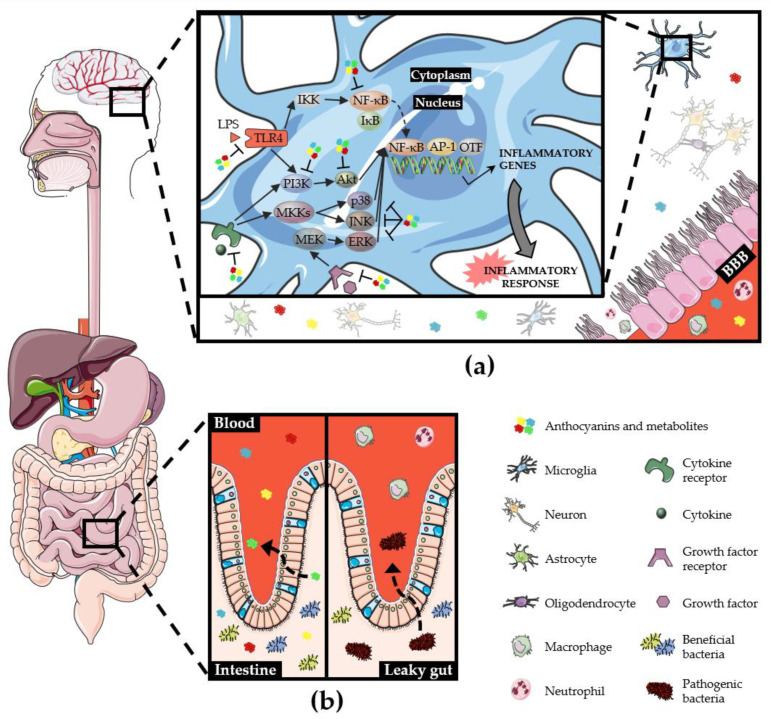
Schematic overview of regulation of microbiota–gut–brain axis by anthocyanins and their metabolites. (**a**) Peripheral inflammatory cells infiltrate into the brain, eliciting microglia activation which involves several inflammatory signaling pathways that can be modulated by anthocyanins and their metabolites; (**b**) Anthocyanins can be absorbed in the intestine, where they firstly promote the proliferation of beneficial bacteria and contribute to the elimination of pathogenic bacteria. The dysregulation of gut microbiota (dysbiosis) or the increase in the intestinal pathogenic community can lead to the disruption of gut wall mucosa (leaky gut), inciting a systemic inflammation and intensifying the neuroinflammatory response. Akt: Protein kinase B; AP-1: Activator protein 1; BBB: Blood–brain barrier; ERK: Extracellular signal-regulated kinase; IĸB: NF-ĸB inhibitor; IKK: IĸB kinase; JNK: c-Jun N-terminal kinase; LPS: Lipopolysaccharide; MEK: Ras/Raf/mitogen-activated protein kinase kinase; MKKs: Mitogen-activated protein kinase kinase; NF-ĸB: Nuclear factor kappa B; OTF: Other transcription factors; p38: Mitogen-activated protein kinase p38; PI3K: Phosphoinositide 3-kinase; TLR4: Toll-like receptor 4.

The etiology of numerous neurological disorders remains undisclosed, although evidence suggests that systemic inflammation is involved in their onset and development [170,171,172]. In fact, the neuroimmune response often encompasses the infiltration of peripheral immune cells, which exacerbates the neuroinflammatory process [77]. Recently, several researchers have postulated that the systemic inflammation occurring in peripheral immune system can amplify neuroinflammation through the connection between the gastrointestinal tract and the brain, known as vagovagal reflex or gut-brain axis [173]. Moreover, gut inflammation and oxidative stress contribute to the disruption of the gut wall mucosa (leaky gut) and the BBB, which allow the entrance of peripheral immune cells, inflammatory cytokines, pathogens, and endotoxins in the brain [172] (Figure 3).

The gastrointestinal tract houses a vast community of bacterial cells that constitute the gut microbiome, whose variable composition is greatly susceptible to diverse external factors, including diet and antibiotics [174]. Accumulating data have revealed the influence of dietary habits in the development of metabolic alterations. Since bacteria compete for nutrients, these dietary constituents can affect the microbiome composition, the production of microbial by-products and the activation of signaling pathways in the gastrointestinal tract [175]. These biological changes may potentiate the production of pro-inflammatory mediators and trigger immune cells activation, partially being responsible for the development of neuroinflammatory conditions and facilitating lower neurological outcomes [175]. Indeed, it has been shown that alterations in gut microbiota composition may induce systemic inflammation with detrimental effects on the CNS [172,176]. Therefore, diets enriched in fruits and vegetables, with significant amounts of antioxidant and anti-inflammatory compounds, could modulate gut microbiota composition, improve systemic metabolic dysfunctions and eventually interfere with multiple cell signaling pathways potentially involved in brain pathologies. It is worthy of note that anthocyanins have been shown to influence the relative composition of gut microbiota, contributing to the maintenance and proliferation of beneficial bacteria population and helping to eradicate pathogenic bacteria [177], thus demonstrating remarkable prebiotic properties (Figure 3). An in vitro study conducted by Zhang et al. revealed that human intestinal microbiota treated with anthocyanins, obtained from purple sweet potato, showed an increase in the levels of beneficial bacteria, such as Lactobacilli and Bifidobacteria [178]. Similarly, an extract of black rice anthocyanins and a purified C3G sample promoted the in vitro growth of Bifidobacteria and Lactobacilli [179]. These microorganisms are considered the predominant intestinal bacteria in humans and act as probiotics, presenting health benefits through their participation in digestion, stimulation of immune response and inhibition of pathogens growth [180]. Another study corroborates the ability of anthocyanins, isolated from purple sweet potato, in stimulating Bifidobacteria growth, accompanied by the production of various phenolic acids [181]. Likewise, peonidin-based anthocyanins extracted from purple sweet potato induced not only the proliferation of Bifidobacteria and Lactobacilli but also the growth inhibition of the intestinal pathogens *Staphylococcus aureus* and *Salmonella typhimurium*, suggesting a prebiotic-like activity of anthocyanins through the modulation of gut microbiota [182]. On the other hand, intestinal bacteria have been described to be able to increase anthocyanin bioavailability through the synthesis of long-lived anthocyanin metabolites, which can be advantageous for potential therapeutic purposes [177,183] (Figure 3). The taxonomic and metabolic heterogeneity of gut microbiota allow the production of a particular metabolite by a specific or various bacterial species that might become a metabolic substrate for other bacterial species [184]. Even though the establishment of correlations between a certain metabolite and a particular bacterial specimen is challenging, new efforts have been made to identify putative associations between microbial metabolites and fecal bacteria, attempting to dissect the gut bacteria species predominantly involved in the levels of a particular polyphenol metabolite [184]. From a therapeutic perspective, these approaches might be useful while studying the beneficial effect of a specific anthocyanin metabolite, since appropriate information about the putative, specific gut microbiota-polyphenols interplay can be drawn.

Since gut microbiome seems to be a critical element in the communication between the gastrointestinal system and CNS, the ability of anthocyanins to modulate microbiota–gut–brain axis needs to be further scrutinized. Several studies have already unveiled the polyphenols effect on microbiota–gut–brain connection in the context of different neurological diseases [58,173,174,185,186,187,188]. Ho et al. showed the impact of the diversity of gut bacteria on the synthesis of flavonoid metabolites, which may affect the alpha-synuclein misfolding, a crucial process in PD onset, and the development of motor dysfunction in a *Drosophila* model of α-synucleinopathy [185]. In fact, the metabolic benefits of anthocyanins extracted from black currants are dependent on the presence of an intact gut microbiota [189], demonstrating the crucial interaction between dietary anthocyanins and intestinal bacteria. Also, 3-hydroxycinnamic acid derived from successive anthocyanin metabolic events has relevant anti-depressive effect, eventually through anti-inflammatory mechanisms [190]. Importantly, a blackberry anthocyanin-rich extract attenuates the neurologic complications of obesity through the modulation of the gut microbiota composition, with the consequent impact on tryptophan metabolism and on the release of kyanurenic acid, a neuroprotective metabolite [188]. These results suggest that dietary manipulation of the gut microbial environment, via anthocyanins administration, could delay or restrict the progression of several CNS disorders. In summary, the neuroprotection can be promoted by the ability of anthocyanins: (1) To abrogate systemic inflammation through the reduction of peripheral inflammatory mediators; (2) to maintain the gastrointestinal function, regulating the gut microbiota and preventing gut inflammation and oxidative stress; (3) to regulate endothelial cell function, preserving or restoring BBB integrity; and (4) to enter the brain and exert their beneficial effect directly on glial cells, controlling neuroinflammatory responses [173,186].

Despite the growing number of studies focusing on the potential interaction between anthocyanins and the microbiota–gut–brain axis, inconsistent data have been reported, particularly concerning the fundamental metabolic pathways and the identification of individual or combined polyphenols that effectively influence metabolism. Since the development of several neurological disorders seems to be strongly influenced by the connection between microbiota and gut-brain axis, mechanistic, in-depth approaches are required to extensively analyze the anthocyanins effect on this critical relationship.

## 6. Conclusions and Future Research

The present review summarizes the main findings in the context of the anti-neuroinflammatory role of anthocyanins in several neuropathological conditions, via modulation of important inflammatory signaling pathways. It should be noted that the activity of anthocyanins hugely depends on the gut microbiota activity, which is engaged in crucial metabolic processes that are critical to increase the anthocyanin bioavailability. In this review, data have been collected demonstrating that anthocyanin metabolites present multiple beneficial properties, such as anti-inflammatory, antioxidant and antiapoptotic functions, being useful as a novel therapeutical strategy for several brain disorders.

Despite anthocyanins have been considered promising therapeutical agents in the context of many neurological disorders, several challenging aspects about anthocyanin bioavailability and about their action mechanisms should be further addressed before clinical use can be recommended. Until this moment, the great majority of reports aiming to study the potential of anthocyanins in the prevention and treatment of brain disorders have been using anthocyanin-rich extracts, usually obtained from anthocyanin-rich fruits, vegetables, or beverages. However, polyphenolic extracts contain a multitude of bioactive micronutrients, including other phenolic compounds beyond anthocyanins, which may also contribute to disease prevention or amelioration. Consequently, it might be difficult to predict whether a specific beneficial effect is owed to a particular anthocyanin. For this reason, the synthesis of pure anthocyanins for research purposes remains relevant to scrutinize the direct correlation between anthocyanin compounds and their bioactivities and to further elucidate their therapeutic efficacy in pre-clinical studies. Nevertheless, potential benefits of the association of various anthocyanins or their combination with other bioactive compounds still remain a matter of debate. On the other hand, researchers should develop improved techniques to evaluate gut microbiota impact on the bioactivity of anthocyanins. For instance, current in vitro approaches cannot faithfully reproduce the interindividual differences of the bacterial population of the human gut. Additionally, the bioactivity of anthocyanin metabolites has been often undervalued and should be carefully considered for the recognition of the beneficial effects of anthocyanins. Finally, further research in the formulation of anthocyanins could be useful to achieve a controlled and targeted release of these compounds, substantially increasing their bioavailability and their potential therapeutic role.

## Figures and Tables

**Table 1:** Summary of the in vitro studies showing the anti-neuroinflammatory role of anthocyanins and their metabolites. I^−^ = inhibition; ↓ = reduction; ↑ = increase.

Anthocyanin/Metabolite	Cells	Anthocyanin Dose	Time of Anthocyanin Exposure	Stimuli/Trigger	Anti-Neuroinflammatory Effect	Ref.
Anthocyanin-rich açai fruit pulp fraction	BV-2 microglial cells	25–1000 μg/mL	4 h	LPS	I^−^ p38-MAPK and NF-κB pathways; ↓ iNOS and COX-2 expressions; ↓ TNF-α production	[28]
Anthocyanin-loaded polyethylene glycol-gold nanoparticles	BV-2 microglial cells	0.1 mg/mL	4 h	Aβ	↓ p-NF-κB, iNOS, COX-2, TNF-α, IL-1β and NOS3 levels	[64]
Anthocyanins-loaded PLGA-PEG nanoparticles	SH-SY5Y cell line	200 μg/mL	12 h	Aβ	↓ p-P38 and p-JNK expressions; ↓ p-NF-κB, TNF-α and iNOS levels	[68]
Anthocyanins (black soybean)	BV-2 microglial cells	50 or 100 μg/mL	1 h	LPS	I^−^ PI3K/Akt, MAPKs and NK-κB pathways; ↓ iNOS and COX-2 expressions; ↓ NO, PGE2, TNF-α and IL-1β production; ↓ NF-κB p65 nuclear level; ↓ IκBα degradation	[99]
Callistephin	C8-4B microglial cells	100 µM	24 h	LPS/IFN-γ	↓ iNOS, COX-2 and TNF-α expressions; ↑ NF-ĸB p65 expression; ↓ iNOS and COX-2 production; ↓ p38 phosphorylation	[100]
Cyanidin-3-O-glucoside (C3G)	BV-2 microglial cells	2.5, 5 or 10 μM	4 h	LPS	I^−^ NF-κB and p38 pathways; ↓ microglial activation; ↓ iNOS, COX-2, IL-1β and IL-6 expressions; ↓ NO, PGE2, IL-1β and IL-6 production	[101]
Protocatechuic Acid	BV-2 microglial cells	5, 10 or 20 μM	24 h	LPS	I^−^ MAPKs and NF-κB pathways; ↓ TNF-α, IL-6, IL-1β, and PGE2 production; I^−^ NF-κB p65 and IκBα phosphorylation; ↓ TLR4 expression	[102]
	BV-2 microglial cells	10, 25, 50 or 100 μM	24 h	LPS	↓ NO production	[103]
Cyanidin; C3G and Methyl-C3G	N9 microglia cell line	1 μM	24 h	LPS	↓ TNF, IL-6 and IL-1β expressions	[104]
Gallic acid	BV-2 or primary microglial cells	5–50 μM	12 h	Aβ	I^−^ NF-κB hyperacetylation; ↓ iNOS, COX-2, TNF-α and IL-1β expressions	[105]
Delphinidin	C6 glial cells	1, 10, 50 or 100 μg/mL	18 h	TNF-α	↓ MCP-1 and CINC-1 levels	[106]
Gallic acid (GA) and Vanillic acid (VA)	Glial cells and hippocampal neurons co-culture	1.0 µM (GA) or 0.2 µM (VA)	72 h	LPC	↓ COX-2 and NF-κB expressions; ↓ astrocyte activation	[107]

**Table 2:** Summary of the in vivo studies showing the anti-neuroinflammatory role of anthocyanins and their metabolites. I^−^ = inhibition; ↓ = reduction; ↑ = increase; — = not evaluated.

Anthocyanin/Metabolite	Anthocyanin Dose	Time of Anthocyanin Exposure	Animal Strain	Animal Model	Behavioral Effect	Brain Region Analyzed	Anti-Neuroinflammatory Effect	Ref.
Anthocyanins (*Vaccinium myrtillus* L.)	30 or 100 mg/kg	10 days	C57BL/6 mice	LPS treatment	Prevented the memory impairment	Cortex and hippocampus	↓ IL-1β and TNF-α production; ↑ IL-10 production; ↓ inflammatory cells infiltration; ↓ microglia and astrocyte activation	[27]
Anthocyanin-loaded polyethylene glycol-gold nanoparticles	10 mg/kg	14 days	C57BL/6 mice	Alzheimer’s disease	—	Cortex and hippocampus	↓ p-NF-κB, iNOS, COX-2, TNF-α, IL-1β and NOS3 levels; ↓ microgliosis and astrocytosis	[64]
	12 mg/kg	14 days	C57BL/6 mice	Alzheimer’s disease	Attenuated memory deficits	Hippocampus	Regulation of p-PI3K and p-Akt levels	[69]
Gallic acid	10 or 30 mg/kg	28 days	ICR mice	Alzheimer’s disease	Attenuated memory and learning impairments	Cortex, hippocampus or whole brain	↓ NF-κB hyperacetylation and nuclear translocation; ↓ iNOS, COX-2, IL-1β and TNF-α production	[105]
Anthocyanins (Korean black soybean)	24 mg/kg	14 days	C57BL/6 mice	LPS treatment	—	Cerebral cortex	↓ NF-κB activation; ↓ IL-1β, TNF-α and COX-2 levels; ↓ microglia and astrocyte activation	[108]
	24 mg/kg	14 days	C57BL/6 mice	LPS treatment	Improved the hippocampus-dependent memory	Hippocampus	↓ IL-1β, TNF-α and p-NF-κB levels	[109]
	100 mg/kg	7 weeks	Sprague-Dawley rat	D-galactose aging model	Reversed memory impairment	Cortex and hippocampus	↓ iNOS, TNF-α and p-NF-κB levels; ↓ microglia and astrocyte activation	[110]
Anthocyanins (*Lycium ruthenicum* Murr)	50–200 mg/kg	7 weeks	Sprague-Dawley rat	D-galactose aging model	Reversed memory impairment	Hippocampus	↓ p-JNK activation; ↓ NF-κB, IL-1β, COX-2 and TNF-α levels; ↓ microglia and astrocyte activation;	[111]
Protocatechuic acid	0.5%, 1% or 2% PCA diet	8 weeks	BALB/c mice	D-galactose aging model	—	Whole brain	↓ NF-κB activation; ↓ COX-2, IL-1β, IL-6, TNF-α and PGE2 levels	[112]
Anthocyanins (*Lycium ruthenicum* Murr)	50 or 100 mg/kg	5 weeks	CD-1 mice	Postoperative cognitive disorder	Improved learning and memory	Hippocampus	I^−^ JNK and p38 MAPK pathways; I^−^ MLK3 activation; ↓ TNF-α, IL-1β and IL-6 expressions; ↓ microglia activation	[113]
Anthocyanins (*Vaccinium myrtillus* L.)	20 mg/kg	3 months	APP/PSEN1 double transgenic mice	Alzheimer’s disease	Improved learning and memory	Hippocampus	↓ TNF-α, NF-κB, IL-1β, IL-6, COX-2, iNOS, CD33 and CX3CR1 expressions; ↑ TLR2, TLR4, TREM2 and TYROBP expressions; regulation of microglia and astrocytes activity	[114]
Protocatechuic acid	100 mg/kg	4 weeks	APP/PSEN1 double transgenic mice	Alzheimer’s disease	Improved learning and memory	Whole brain	↓ TNF-α, IL-1β, IL-6 and IL-8 levels	[115]
Anthocyanins (purple sweet potato)	350 or 700 mg/kg	4 weeks	C57BL/6 mice	LPS treatment	Reversed motor and exploration behavior impairments; improved learning and memory	Whole brain	I^−^ p-ERK, p-JNK and NF-κB pathways; ↓ COX-2, iNOS, IL-1β, IL-6 and TNF-α levels	[116]
Anthocyanin (grape skin)	30 or 100 mg/kg	7 days	Wistar rat	Demyelination model	—	Pons	↓ IL-1β, IL-6, TNF-α and IFN-γ levels; ↓ inflammatory cells infiltration; ↑ IL-10 production	[117]
C3G (*Myrica rubra*)	100, 150 or 300 mg/kg	7 days	ICR mice	Ischemic stroke model	—	Cerebral cortex	↓ TLR4, TNF-α, IL-18 and NLRP3 levels; ↑ Nrf2 levels	[118]
Vanillic acid	100 mg/kg	14 days	Wistar rat	Ischemic stroke model	Restored memory impairment	Hippocampus	↓ IL-6 and TNF-α levels; ↑ IL-10 levels	[119]
Pentunidin-3-O-rutinoside (p-coumaroyl)-5-O-glucoside	200 mg/kg	7 days	Sprague–Dawley rat	Ischemic stroke model	Attenuated cognitive function decline	Cerebral cortex	I^−^ NF-κB and NLRP3 inflammasome pathways; ↓ TNF-α, IL-1β and IL-6 production	[120]

## Abbreviations

AD	Alzheimer’s disease
Akt	Protein kinase B
AP-1	Activator protein 1
Aβ	Amyloid beta peptide
BBB	Blood–brain barrier
C3G	Cyanidin-3-O-glucoside
CNS	Central nervous system
COX-2	Cyclooxygenase-2
DG	D-galactose
ERK	Extracellular signal-regulated kinase
GA	Gallic acid
HBA	4-hydroxybenzoic acid
IFN	Interferon
IKK	IκB kinase
IL	Interleukin
iNOS	Nitric oxide synthase
JAK	Janus kinase
JNK	c-jun N-terminal kinase
LPS	Lipopolysaccharide
MAPK	Mitogen-activated protein kinase
MLK3	Mixed-lineage protein kinase 3
MS	Multiple sclerosis
mTOR	Mammalian target of rapamycin
NF-κB	Nuclear factor kappa B
NLRP3	NLR family pyrin domain-containing protein 3
NO	Nitric oxide
PA	Protocatechuic acid
PD	Parkinson’s disease
PGE2	Prostaglandin E2
PI3K	Phosphoinositide 3-kinase
PND	Perioperative neurocognitive disorder
ROS	Reactive oxygen species
STAT	Signal transducer and activator of transcription
TLR	Toll-like receptor
TNF	Tumor necrosis factor
VA	Vanillic acid
